# Undernutrition, Vitamin A and Iron Deficiency Are Associated with Impaired Intestinal Mucosal Permeability in Young Bangladeshi Children Assessed by Lactulose/Mannitol Test

**DOI:** 10.1371/journal.pone.0164447

**Published:** 2016-12-01

**Authors:** Md. Iqbal Hossain, Rashidul Haque, Dinesh Mondal, Mustafa Mahfuz, AM Shamsir Ahmed, M. Munirul Islam, Richard L. Guerrant, William A. Petri, Tahmeed Ahmed

**Affiliations:** 1 Nutrition and Clinical Services Division (NCSD), International Centre for Diarrhoeal Disease Research, Bangladesh (icddr,b), Mohakhali, Dhaka, Bangladesh; 2 James P. Grant School of Public Health, Brac University, Mohakhali, Dhaka, Bangladesh; 3 Division of Infectious Disease and International Health, University of Virginia, Charlottesville, Virginia, United States of America; The Hospital for Sick Children, CANADA

## Abstract

**Background:**

Lactulose/mannitol (L:M) test has been used as a non-invasive marker of intestinal mucosal -integrity and -permeability (enteropathy). We investigated the association of enteropathy with anthropometrics, micronutrient- status, and morbidity in children.

**Methods:**

The urine and blood samples were collected from 925 children aged 6–24 months residing in Mirpur slum of Dhaka, Bangladesh during November 2009 to April 2013. L:M test and micronutrient status were assessed in the laboratory of International Centre for Diarrhoeal Diseases Research, Bangladesh (icddr,b) following standard procedure.

**Results:**

Mean±SD age of the children was 13.2±5.2 months and 47.8% were female. Urinary- lactulose recovery was 0.264±0.236, mannitol recovery was 3.423±3.952, and L:M was 0.109±0.158. An overall negative correlation (Spearman’s-rho) of L:M was found with age (r_s_ = -0.087; p = 0.004), weight-for-age (r_s_ = -0.077; p = 0.010), weight-for-length (r_s_ = -0.060; p = 0.034), mid-upper-arm-circumference (r_s_ = -0.098; p = 0.001) and plasma-retinol (r_s_ = -0.105; p = 0.002); and a positive correlation with plasma α-1-acid glycoprotein (r_s_ = 0.066; p = 0.027). However, most of the correlations were not very strong. Approximately 44% of children had enteropathy as reflected by L:M of ≥0.09. Logistic regression analysis revealed that younger age (infancy) (adjusted odds ratio (AOR) = 1.35; p = 0.027), diarrhea (AOR = 4.00; p = 0.039) or fever (AOR = 2.18; p = 0.003) within previous three days of L:M test were the risk factors of enteropathy (L:M of ≥0.09).

**Conclusions:**

Enteropathy (high L:M) is associated with younger age, undernutrition, low vitamin A and iron status, and infection particularly diarrhea and fever.

## Introduction

Environmental enteropathy is a disorder often subclinical that usually takes place among the residents of low-and middle income (developing) countries where the sanitation is often poor and hygiene practice is sub optimal. Persistent contact/exposure to fecal pathogens may trigger inflammation and structural changes in the small bowel, which ultimately result in functional changes. It is manifested by increased intestinal permeability, malabsorption, growth faltering in individuals without overt diarrhea [1, 2]. It was reported that severely malnourished children have impaired intestinal mucosal function compared with better nourished children from the same environments [3, 4]. It was also found that multiple micronutrient supplementation improved small bowel villous height and absorptive area in adults [5]. This kind of enteropathy is presumed to be common in Bangladesh and can be assessed by Lactulose and Mannitol intestinal permeability test.

The Lactulose/Mannitol intestinal permeability test (L:M) is a non-invasive technique to measure small intestinal epithelial area, transcellular and paracellular transport, damage and permeability. L:M is considered as a consistent and sensitive marker of intestinal permeability [6], and it has been used as an indicator of intestinal mucosal integrity and function [7, 8]. Absorption and urinary excretion of two orally administered non-metabolizable sugars, such as lactulose and mannitol are measured and their ratio is L:M. Lactulose is assumed to permeate through the tight junctions and extrusion zones of the intervillous spaces and mannitol enters the mucosal cell through the hydrophilic portion of the cell membrane. Consequently, lactulose absorption (as assessed by urinary excretion) can be considered as a measure of the integrity of intestinal mucosal tight junctions, and mannitol absorption as an indicator of the mucosal absorptive area, [9]. Thus both aspects of intestinal mucosal function can be assessed, and the use of their urinary recovery ratio provides the additional advantage of controlling for extraneous factors such as gastric retention, liver disease, or partial intra-intestinal or urinary bacterial degradation of the sugars, which may otherwise confound the test results [10, 11]. The L:M was found impaired during acute and chronic disorders of the small intestine, and probably in malnutrition. There is insufficient information on L:M and its association with micronutrient status plus markers of inflammation. We investigated the association of intestinal mucosal -integrity and -permeability (enteropathy) with nutritional- and micronutrient- status, and morbidity in children.

## Methods

From November 2009 to April 2013, 500 underweight (weight-for-age z-score (WAZ) <-2) and 480 well-nourished (WAZ >-1) children aged 6–24 months were enrolled in a study, in Mirpur slum of Dhaka, Bangladesh to find the interactions of enteric infections and malnutrition [12]. On enrollment L:M, short term morbidity (fever and/or diarrhea within last three days), and biochemical data were available of 925 (483 underweight and 442 well-nourished) children, and were analyzed.

### Study site

The study was conducted among residents of an under-privileged community in Mirpur, Dhaka, Bangladesh. Mirpur is inhabited by poor and middle class families, residential and sanitary conditions are typical of any congested urban settlement and it is one of the 14 thanas (sub-district) of Dhaka City with a population of about one million in an area of 59 square kilometers. About 75% of fathers and 85% of mothers were illiterate or had less than 5 years of formal education. The average family income was Tk.4200 (about US $68) per month.

### Data collection

Research assistant interviewed the mother/caregiver using a pretested, structured questionnaire and took morbidity data for last three days and did the anthropometrics. Children’s nude weight using a frequently-standardized digital scale with 10 g precision (Seca, model-345, Hamburg, Germany), Mid-upper-arm-circumference (MUAC) and recumbent length were obtained to the nearest mm using standard procedures. The new WHO growth standard (2006) was used to calculate different anthropometric indices: weight-for-age z-score (WAZ), length-for-age z-score (LAZ) and weight-for-length z-score (WLZ).

### Procedure of L:M test

Mothers were informed earlier, and they did not offer any food to the children on the test day for approximately 2 hours before ingesting a test solution, that contained lactulose (250 mg/ml) and mannitol (50 mg/ml) in a dose of 2 ml/Kg of weight or a maximum 20 ml. The children were requested to empty their bladders whenever possible while fasting and before ingesting the test sugar solution. A pediatric urine collection bag was attached to children to collect their urine. The children were allowed for their regular diet 30 minutes after ingestion of the lactulose-mannitol test solution and urine was collected for five hours, during which time the caregiver offered children liquids frequently in order to permit collection of an adequate volume of urine. For every 50 mL of urine 1–2 drops of 2.35% chlorhexidine is added as a preservative during collection. Total urine volume during these five hours was measured and recorded and a sample of five ml was stored in a -80°C freezer until laboratory analysis.

Laboratory procedures: Lactulose and mannitol were measured using High Performance Anion-Exchange Chromatography with Pulsed Amperometric Detection (HPAE-PAD) [13, 14]. In this method we employed Thermo Scientific Dionex ICS-3000 with CarboPac-MA1 column for the determination of weakly ionized sugars in high concentrations of sodium hydroxide. At high pH, carbohydrates are electro catalytically oxidized at the surface of the gold electrode by application of a positive potential. The current generated is proportional to the carbohydrate concentration, and therefore carbohydrates can be detected and quantified.

After the samples were vortex-mixed for 10 seconds and centrifuged for 10 minutes @ 4500 rpm, supernatant were collected and diluted 30-fold with de-ionized water and internal standard (melibiose) to a volume of 3 mL. The solutions were mixed thoroughly and 1 mL aliquots of sample were transferred into a 1.5 mL injection vial. All samples were automatically injected to the HPIC (Dionex ICS-3000 Ion Chromatography (IC) with auto sampler AS 40, Detector ICS-3000 DC) and results were automatically calculated from the standard curve.

For quality control purposes, following controls were used: (i) pooled urine, (ii) standard containing both lactulose (0.75mM) and mannitol (0.75mM), and (iii) spiked urine. Pooled urine: urine samples were collected from 20 individuals, pooled and used for assessment of sugars; target values were set and the pooled sample was used with every analysis. Spiked urine: recovery was determined by adding known amounts of sugars to urine and comparing the concentration of sugar estimated with the amount added. The results ranged from 93% to 105% for lactulose and 93% 100% for mannitol for spiked urine. Co-efficient of variation (CV %) was 5.3% for lactulose and 2.8% for mannitol in pooled urine respectively. CV% was 1.3% for lactulose and 2.6% for mannitol in the known standards.

The recovery of each sugar excreted in the urine (%) was then calculated using the total urine volume and individual sugar concentrations.

Calculation of urinary lactulose and mannitol recovery and their ratio:

Urinary lactulose recovery = [(total urine volume (dL) over 5 hours multiplied by lactulose in mg/dL of urine) multiplied by 100] divided by amount (mg) of lactulose taken.

Urinary mannitol recovery = [(total urine volume (dL) over 5 hours multiplied by mannitol in mg/dL of urine) multiplied by 100] divided by amount (mg) of mannitol taken.

Urinary lactulose by mannitol recovery ratio = urinary lactulose recovery divided by urinary mannitol recovery.

### Ethical consideration

The study (including the option of taking written consent from the guardians) was approved by the Research Review Committee and Ethical Review Committee of icddr,b. Written consent was given by the mothers or legal guardians of the children. They were made aware about the voluntary participation and informed about the study and had the rights for non-response to the questionnaires or other study related activities. They retained the rights to withdraw their children from the study at any time. All information, research data, and related records were anonymously entered in computer and analyzed without indicating the child’s name and identity. All the investigators completed the “Human Participants Protection Education for Research Teams” online course, sponsored by the National Institutes of Health.

### Data analysis

Data were entered using SPSS for Windows (version 11.5; SPSS Inc., Chicago, IL, USA), and analyzed using SPSS and WHO Anthro software (Geneva, Switzerland). For normally distributed continuous variables, means were compared using unpaired t-tests after checking the equality of variance (Levene's test). For non-normally distributed continuous variables, the Mann-Whitney U test was performed. The association/relation between continuous variables was checked by correlation analysis (Spearman’s-rho for non-parametric data). Differences in proportions were compared by the Chi-square test or Fisher’s exact test if the expected number in any cell was < 5. All independent variables were analyzed initially in bivariate models and the attributes that were significantly associated with enteropathy (L:M of ≥ 0.09) (dependent variable) and biologically plausible were included in logistic regression models with stepwise backward elimination. A probability of less than 0.05 was considered statistically significant. The strength of association was determined by estimating the odds ratios (OR) and their 95% confidence intervals (95% CI).

## Results

Mean±SD age of the children was 13.2±5.2 months, WAZ was -1.60±1.28, LAZ was -1.69±1.23, WLZ was -0.95±1.16, MUAC was 13.8±1.2cm, and 47.8% were female (Table 1). Urinary lactulose recovery was 0.264±0.236%, mannitol recovery was 3.423±3.952%, and L:M was 0.109±0.158, and approximately 44% of the children had an L:M > 0.09 (Table 1). Hemoglobin was 10.7±1.3 g/dl, α-1-acid glycoprotein (AGP) was 100±39 mg/dl, plasma retinol was 25.7±15.0 μg/dl, plasma zinc was 0.75±0.13mg/L, vitamin D was 58.4±25.6 nmol/L, plasma-ferritin was 26.6±33.5 ng/ml, and plasma-transferrin was 7.11±4.07 μg/ml (Table 1).

**Table 1:** Age, sex, anthropometric characteristics, lactulose and mannitol (L:M) test results and other biochemical values among the studied children.

	Children altogether N = 925
Age (month)	13.1±5.2
Female: n (%)	442 (47.8)
Weight	7.93±1.39
Length/Height (cm)	71.3±5.6
Weight-for-age z-score	-1.60±1.28
Height-for-age z-score	-1.69±1.23
Weight-for-height z-score	-0.95±1.16
Mid upper arm circumference (cm)	13.8±1.2
Urinary lactulose recovery (%) [median (interquartile range)]	0.264±0.236 [0.206 (0.110, 0.349)]
Urinary mannitol recovery (%) [median (interquartile range)]	3.423±3.952 [2.559 (1.479, 4.321)]
Ratio of urinary recovery of lactulose and mannitol (L:M) [median (interquartile range)]	0.109±0.158 [0.081(0.050, 0.129]
L:M ≥ 0.09: n (%)	406 (43.9)
α-1-acid glycoprotein (mg/dl): Mean±SD) [median (interquartile range)]	100±39 [93 (72, 122)]
Hemoglobin (g/dl)	10.7±1.3
Plasma-retinol(μg/dl) [median (interquartile range)]	25.7±15.0 [22.2 (17.8, 28.5)]
Plasma zinc (mg/L)	0.75±0.13
Vitamin D (nmol/L)	58.4±25.6
Plasma-ferritin (ng/ml) [median (interquartile range)]	26.6±33.5 [17.9 (9.2, 32.2)]
Plasma-transferrin (μg/ml) [median (interquartile range)]	7.11±4.07 [5.93 (4.69, 8.16)]

Data are mean±SD if not mentioned otherwise

Table 2 shows the correlation (Spearman’s-rho) of different anthropometric and biochemical characteristics and urinary recovery of lactulose and mannitol, and L:M in all children (among underweight- and well-nourished altogether). However, most of the correlations observed were not very strong.

**Table 2:** Overall (among underweight- and well-nourished- children altogether) correlations (Spearman’s-rho) between lactulose/mannitol (L:M) test.

Variable	Age	WHZ	HAZ	WAZ	MUAC	AGP	Hb	Zinc	Vitamin D	p Ferritin	p Retinol	P TfR
	*n-925*	*n-925*	*n-925*	*n-925*	*n-922*	*n-867*	*n-758*	*n-976*	*n-835*	*n-816*	*n-799*	*n-868*
Urinary lactulose recovery	-0.014	-0.025	0.019	-0.011	-0.028	**0.146**	-0.047	**0.084**	0.003	**-0.128**	0.020	**0.103**
	0.336	0.221	0.279	0.365	0.196	**0.036**	0.198	**0.017**	0.467	**<0.001**	0.288	**0.001**
Urinary mannitol recovery	**0.071**	0.045	**0.065**	**0.073**	**0.070**	-0.007	-0.046	0.013	-0.052	**-0.091**	**0.124**	0.054
	**0.016**	0.086	**0.024**	**0.013**	**0.017**	0.414	0.206	0.706	0.066	**0.005**	**<0.001**	0.054
L:M	**-0.087**	**-0.060**	-0.039	**-0.077**	**-0.098**	**0.066**	-0.070	0.062	0.056	-0.021	**-0.105**	0.053
	**0.004**	**0.034**	0.115	**0.010**	**0.001**	**0.027**	0.052	0.083	0.052	0.276	**0.002**	0.061

WLZ = weight-for-length z-score, LAZ = length-for-age z-score, WAZ = weight-for-age-z score, MUAC = mid upper arm circumference, AGP = α-1-acid glycoprotein, Hb = hemoglobin, p TfR = plasma-transferrin

First row of each variable is Spearman’s-rho; second row of each variable is the related p value

Lactulose recovery: a negative correlation between plasma-ferritin (r_s_ = -0.128; p<0.001) and positive correlation between plasma-transferrin (r_s_ = 0.103; p = 0.001) was observed with urinary lactulose recovery.

Mannitol recovery: a negative correlation between plasma ferritin (r_s_ = -0.091; p = 0.005) and positive correlation was found between age (r_s_ = 0.071; p = 0.016), WAZ (r_s_ = 0.073; p = 0.013), LAZ (r_s_ = 0.065; p = 0.024), MUAC (r_s_ = 0.070; p = 0.017) and plasma-retinol (r_s_ = 0.124; p<0.001) with urinary mannitol recovery.

Urinary L:M: a negative correlation of L:M was found with age (r_s_ = -0.087; p = 0.004), WAZ (r_s_ = -0.077; p = 0.010), WLZ (r_s_ = -0.060; p = 0.034), MUAC (r_s_ = -0.098; p = 0.001) and plasma-retinol (r_s_ = -0.105; p = 0.002); and a positive correlation was found with AGP (r_s_ = 0.066; p = 0.027).

Approximately 44% of children had enteropathy as reflected by L:M of ≥0.09 (Table 2). Table 3 shows the distribution of age, sex, nutritional status, biochemical characteristics, and morbidities as categorical variables are shown by enteropathy (L:M of ≥0.09) in bivariate analysis. Logistic regression analysis revealed that younger age (infancy) (adjusted odds ratio (AOR) = 1.35; p = 0.027), having diarrhea (AOR = 4.00; p = 0.039) or fever (AOR = 2.18; p = 0.003) within previous three days of lactulose/mannitol test were the associated/risk factors of enteropathy (Table 4).

**Table 3:** Age, sex, nutritional status, biochemical characteristics, and morbidities by impaired intestinal mucosal -integrity and -permeability (L:M ≥ 0.09), bivariate analysis.

	L:M ≥ 0.09 N = 406	L:M < 0.09 N = 519	p value
Infant (Age <12 months) on enrollment	211 (52.0)	232 (44.7)	**<0.001**
Sex: female	201 (49.5)	241 (46.4)	0.389
Underweight (weight for age z score < -2)	209 (51.5)	274 (52.8)	0.691
α-1-acid glycoprotein > 100 mg/dl	173 (45.2)	203 (41.9)	0.370
Moderate to severe anemia (Hemoglobin < 10 g/dL)	82 (24.6)	114 (26.9)	0.504
Plasma retinol < 20	138 (38.2)	149 (34.0)	0.236
Plasma zinc < 0.65 mg/L	64 (18.3)	83 (18.6)	0.927
Plasma-ferritin < 12 ng/ml	119 (32.1)	157 (35.3)	0.373
Plasma-transferrin > 8.3 μg/ml	90 (23.4)	118 (24.4)	0.749
History of diarrhea within 3 days of L:M test	9 (2.2)	3 (0.6)	**0.029**
History of fever within 3 days of L:M test	40 (9.9)	25 (4.8)	**0.002**

Differences in proportions were compared by the Chi-square test or Fisher’s exact test if the expected number in any cell was < 5.

**Table 4:** Risk factors of impaired intestinal mucosal -integrity and -permeability (L:M ≥ 0.09) by backward logistic regression analysis.

	L:M ≥ 0.09 N = 406	L:M < 0.09 N = 519	OR (95% CI)of L:M ≥ 0.09	p value
Infant (age <12 months)	211 (52.0)	232 (44.7)	1.35 (1.04, 1.75)	**0.027**
History of diarrhea within 3 days of L:M test	9 (2.2)	3 (0.6)	4.01 (1.07, 14.98)	**0.039**
History of fever within 3 days of L:M test	40 (9.9)	25 (4.8)	2.18 (1.29, 3.66)	**0.003**

Variables entered (only those were significant (p<0.05) in bivariate analysis) in the model: infant (age <12 months); history of diarrhea within 3 days of L:M test; and history of fever within 3 days of L:M test

## Discussion

We investigated the intestinal mucosal -integrity and -permeability (enteropathy) by duel sugar-permeability test and are describing in this paper its association with nutritional- and micronutrient- status, and morbidity in children. We conducted the test to measure the amount of lactulose and mannitol recovered in a 5-hour urine sample as suggested by many investigators in their earlier reports. Permeability to lactulose if increases it indicates leaky gut, while permeability to mannitol if decreases it indicates malabsorption of small molecules. The lactulose/mannitol ratio (LM) is thus considered as a useful parameter, and an elevated ratio indicates impaired-IMIP.

The present study found that the children’s age was directly related with urinary mannitol recovery and inversely related with L:M. We believe that maturation of the intestinal epithelium (thus lager absorptive capacity) takes place with advancement of child’s age and that is the reason of such observation. Previous data have shown association of undernutrition with intestinal villus atrophy and disruption of the intestinal barrier function [15, 16]. Like the above mentioned findings the nutritional status of the children assessed by WLZ, WAZ, and MUAC in the current study was found inversely associated with intestinal permeability results. It is uncertain, however, whether undernutrition was the cause or result of altered small intestinal function. It is recognized that underourished children are more susceptible to infections [17]. In our current study we also observed that AGP and CRP (data not shown) level was directly correlated with undernutrition and L:M. Moreover, studies in experimental animals have found that intestinal mucosal recovery after enteric infection is delayed in malnourished hosts [18]. Abnormality of intestinal permeability may also be associated with malabsorption of macro and micronutrients, thereby causing secondary malnutrition. Field studies in Gambia found that children with abnormal intestinal permeability had slower growth during first year of observation [19]. Thus, it is possible that enteropathy may have contributed to undernutrition in these children. Lunn [20] and Campbell et al., [21] ascribed 64% of height and weight faltering in two to 15 month-old Gambian infants as being due to impaired intestinal integrity and absorption. They suggested that frequent gastrointestinal infections damaged the small intestinal mucosal function, with loss of vulnerable brush-border enzymes, including lactase, thereby impairing digestion and absorption as well as disrupting barrier function, and allowing translocation of macromolecules across the mucosa and into the body.

The current study found a significant inverse correlation between iron status (lower serum ferritin and higher transferrin) and urinary lactulose recovery, confirming the results of previous studies [22, 23]. This suggests that adequate iron reserves may help in maintaining normal intestinal mucosal function. Alternatively, it is conceivable that those intestinal changes as identified by permeability studies may interfere with iron absorption. It should be considered that ferritin is also an acute phase protein and can be increased in the context of inflammation that may explain the positive correlation with urinary mannitol recovery. However, speculation regarding the correlation and its variation in iron binding proteins is difficult to infer without iron concentrations and iron saturation levels (those were not done in this study).

It the current study analyses of plasma retinol level showed a significant direct correlation between urinary mannitol recovery and inverse correlation between urinary L:M ratios and plasma retinol. But no association was observed between zinc and L:M. As it is well known that plasma zinc concentration is a poor indicator of zinc status in individual subjects [24]. Thus, the absence of a significant association between serum zinc concentrations and intestinal permeability study results may not necessarily rule out a functional relationship between zinc status and intestinal mucosal integrity. This observed effect was presumably accounted for predominantly by higher mannitol absorption with higher serum retinol levels. Since mannitol uptake/excretion is an indicator of overall villous surface area, this suggests that either villous surface area is compromised as a result of vitamin A deficiency and/or vitamin A status is compromised in children who have decreased villous surface area. Either possibility, and quite likely both, could be happened. Although one study from Bangladesh [25] found that supplementation of vitamin A and zinc resulted in significant reductions (improvements) in the L:M. The changes in the L:M were largely due to a decrease in lactulose absorption, which might be predominantly an effect of zinc, based on the evidence that the reductions of lactulose excretion, but not increased mannitol absorption, were significantly influenced by zinc supplementation in Bangladeshi children with acute and persistent diarrhea [25]. Thus, vitamin A and zinc may have different types of effect on intestinal permeability that are complementary and may be synergistic. The correlations were not very strong, and that reflects that there would be other factors not captured in the present study, that might hamper the altered lactulose and mannitol permeability.

An upper normal limit of L:M (mean + 2 standard deviations) of 0.09 was found among 30 healthy Dutch children whose ages ranged from 0 to 16 years [26]. We define enteropathy if L:M is ≥0.09 as used by others [13, 27] and found that about 44% of all children had enteropathy. In an earlier study it was found that over 57% of the Bangladeshi infants had abnormal intestinal permeability values throughout the study [28]. Recent history of diarrhea was associated with impaired intestinal integrity and absorption in our study as also found by others [4, 27, 29]. The results further agree with several reports published from the data of children with acute and persistent diarrhea [8, 30–32].

Because diarrhea and/or fever themselves can alter the intestinal permeability, we have reanalyzed the correlations from the data excluding the children having history of previous three days diarrhea and/or fever. The correlation (Spearman’s-rho) between urinary lactulose recovery and: plasma zinc (r_s_ = 0.091; p = 0.014); plasma ferritin (r_s_ = -0.134; p<0.001); and plasma-transferrin (r_s_ = 0.113; p = 0.001); and between urinary mannitol recovery and plasma ferritin (r_s_ = -0.0.85; p<0.020) were found similar to that found before without excluding the data of children having history of diarrhea and/or fever.

In our knowledge we are the first time reporting that recent history of fever is also associated with impaired intestinal integrity and absorption. Although we did not find any correlation with vitamin D level and L:M, but in our knowledge we are the first group who tried to assess this association and reporting about that. In conclusion, this study found that probably due to lack of maturation of the intestinal cellular structure at younger age the L:M is comparatively higher than that when the children are grown up. High L:M is found to be also associated with, undernutrition and low iron status. Lower body iron status is associated with defect in tight junction in intestinal epithelium (as reflected by more para-cellular absorption and higher urinary lactulose recovery). The positive correlation between urinary mannitol recovery, and age and nutritional status reflects maturation of intestinal epithelium takes place with increments of child’s age and better nutritional status, and vitamin A helps the process. Moreover, diarrhea, and fever are also associated/risk factors of impaired-IMIP.

## References

[1] Sullivan PB, Marsh MN, Mirakian R, Hill SM, Milla PJ, Neale G (1991). Chronic diarrhea and malnutrition--histology of the small intestinal lesion. J Pediatr Gastroenterol Nutr 12:195–203. 1904932 10.1097/00005176-199102000-00010

[2] Ramakrishna BS, Venkataraman S, Mukhopadhya A. Tropical malabsorption (2006). Postgrad Med J 82:779–787. 10.1136/pgmj.2006.048579 17148698 PMC2653921

[3] Brewster DR, Manary MJ, Menzies IS, O’Loughlin EV, Henry RL (1997). Intestinal permeability in kwashiorkor. Arch Dis Child 76:236–241. 9135265 10.1136/adc.76.3.236 PMC1717121

[4] Goto K, Chew F, Torun B, Peerson JM, Brown KH (1999). Epidemiology of altered intestinal permeability to lactulose andmannitol in Guatemalan infants. J Pediatr Gastroenterol Nutr 28:282–290. 10067729 10.1097/00005176-199903000-00013

[5] Louis-Auguste J, Greenwald S, Simuyandi M, Soko R, Rose Banda R, Kelly P (2014). High dose multiple micronutrient supplementation improves villous morphology in environmental enteropathy without HIV enteropathy: results from a double-blind randomised placebo controlled trial in Zambian adults. BMC Gastroenterol 14:15 Published online 2014 1 15 10.1186/1471-230X-14-15 24428805 PMC3897937

[6] Menzies IS, Laker MF, Pounder R. Abnormal permeability to sugars in villous atrophy (1979). Lancet 8152:1107–1109. 10.1016/s0140-6736(79)92507-8 91841

[7] Ukabam SO, Homeida MMA, Cooper BT (1986). Small intestinal permeability in normal Sudanese subjects: evidence of tropical enteropathy. Trans R Soc Trop Med Hyg 80:204–207. 3097886 10.1016/0035-9203(86)90010-6

[8] Behrens RH, Lunn PG, Northrop CA, Hanlon PW, Neale G (1987). Factors affecting the integrity of the intestinal mucosa of Gambian children. Am J Clin Nutr 45:1433–1441. 3109230 10.1093/ajcn/45.6.1433

[9] Fleming SC, Kapembwa MS, Laker MF, Levin GE, Griffin GE (1990). Rapid and simultaneous determination of lactulose and mannitol in urine, by HPLC with pulsed amperometric detection, for use in studies of intestinal permeability. Clin Chem 36:797–799. 2110873

[10] Menzies IS (1983). Transmucosal passage of inertmolecules in health and disease. In: Skadhauge E,Heintze K, eds. Intestinal Absorption and Secretion. Falk Symposium 36. Lancaster, UK: MTP Press Ltd 527–543.

[11] Willumsen JF, Darling JC, Kitundu JA, (1997). Dietary management of acute diarrhoea in children: effect of fermented and amylase-digested weaning foods on intestinal permeability. J Pediatr Gastroenterol Nutr 24:235–241. 9138166 10.1097/00005176-199703000-00001

[12] The MAL-ED Network Investigators (2014). The MAL-ED study: a multinational and multidisciplinary approach to understand the relationship between enteric pathogens, malnutrition, gut physiology, physical growth, cognitive development, and immune responses in infants and children up to 2 years of age in resource-poor environments. Clin Infect Dis 59:S193–S206. 10.1093/cid/ciu653 25305287

[13] Bao Y, Silva TMJ, Guerrant RL, Lima AAM, Fox JW (1996). Direct analysis of mannitol, lactulose and glucose in urine samples by high-performance anion-exchange chromatography with pulse amperometric detection: clinical evaluation of intestinal permeability in human immunodeficiency virus infection. Journal of Chromatography B 685:105–112. 10.1016/0378-4347(96)00159-4 8930758

[14] Barboza MS Jr, Silva TMJ. Guerrant RL and Lima AAM (1999). Measurement of intestinal permeability using mannitol and lactulose in children with diarrheal diseases. Braz J Med Biol Res 32:1499–1504. 10585631 10.1590/s0100-879x1999001200008

[15] Sullivan PB, Lunn PG, Northrop-Clewes C, Crowe PT, Marsh MN, Neale G (1992). Persistent diarrhoea and malnutrition: the impact of treatment on small bowel structure and permeability. J Pediatr Gastroenterol Nutr 14:208–215. 1593377 10.1097/00005176-199202000-00016

[16] Lima AA, Brito LF, Ribeiro HB, Martins MC, Lustosa AP, Rocha EM, Lima NL, Monte CM, Guerrant RL. (2005). Intestinal barrier function and weight gain in malnourished children taking glutamine supplemented enteral formula. J Pediatr Gastroenterol Nutr 40:28–35. 15625423 10.1097/00005176-200501000-00006

[17] Brown KH, Solomons NW (1991). Nutritional problems of developing countries. Infect Dis Clin North Am 5:297–317. 1869811

[18] Butzner JD, Butler DG, Mimiats OP, Hamilton JR (1985). Impact of chronic protein-calorie malnutrition on small intestinal repair after acute viral enteritis: A study in gnotobiotic piglets. Pediatr Res 19:476–481. 3923424 10.1203/00006450-198505000-00014

[19] Lunn PG, Northrop-Clewes CA, Downes RM (1991). Intestinal permeability, mucosal injury, and growth faltering in Gambian infants. Lancet 338:907–10. 1681266 10.1016/0140-6736(91)91772-m

[20] Lunn PG (2000). The impact of infection and nutrition on gut function and growth in childhood. Proc Nutr Soc 59:147–154. 10828184 10.1017/s0029665100000173

[21] Campbell DI, Elia M, Lunn PG (2003). Growth faltering in rural Gambian infants is associated with impaired small intestinal barrier function, leading to endotoxemia and systemic inflammation. J Nutr 133:1332–1338. 12730419 10.1093/jn/133.5.1332

[22] Juby LD, Rothwell J, Axon ATR (1989). Lactulose/mannitol test: An ideal screen for celiac disease. Gastroenterology 96:79–85. 2491824 10.1016/0016-5085(89)90767-1

[23] Berant M, Khourie M, Menzies IS (1992). Effect of iron deficiency on small intestinal permeability in infants and young children. J Pediatr Gastroenterol Nutr 14:17–20. 1573506 10.1097/00005176-199201000-00004

[24] Aggett PJ (1990). Malnutrition and trace element metabolism In: The malnourished child. New York: Nestle, Vevey/Raven Press 156–176.

[25] Roy SK, Behrens RH, Haider R, Akramuzzaman SM, Mahalanabis D, Wahed MA, Tomkins AM (1992). Impact of zinc supplementation on intestinal permeability in Bangladeshi children with acute diarrhoea and persistent diarrhoea syndrome. J Pediatr Gastroenterol Nutr 15:289–296. 1432467 10.1097/00005176-199210000-00010

[26] Van Elburg RM, Uil JJ, Kokke FT, Mulder AM, van de Broek WG, Mulder CJ, Heymans HS (1995). Repeatability of the sugar-absorption test, using lactulose and mannitol, for measuring intestinal permeability for sugars. J Pediatr Gastroenterol Nutr 20:184–188. 7714684 10.1097/00005176-199502000-00008

[27] Boaz RT, Joseph AJ, Kang G, Bose A (2013). Intestinal permeability in normally nourished and malnourished children with and without diarrhea. Indian Pediatr 50:152–3. 23396789 10.1007/s13312-013-0030-3

[28] Goto R, Mascie-Taylor CGN, Lunn PG (2009). Impact of anti-Giardia and anthelminthic treatment on infant growth and intestinal permeability in rural Bangladesh: a randomised double-blind controlled study. Trans R Soc Trop Med & Hyg 103:520–529. 18789466 10.1016/j.trstmh.2008.07.020

[29] Brown KH, Parry L, Khatun M, Ahmed G (1979). Lactose malabsorption in Bangladeshi village children: relation with age, history of recent diarrhea, nutritional status, and breast feeding. Am J Clin Nutr 32:1962–1969. 474486 10.1093/ajcn/32.9.1962

[30] Ford RPK, Menzies IS, Philips AP, Walker-Smith JA, Turner MW (1985). Intestinal sugar permeability: Relationship to diarrhea disease and small bowel morphology. J Pediatr Gastroenterol Nutr 4:468–474. 4032170

[31] Isolauri E, Juntunen M, Wiren S, Vuorinen P, Koivula T (1989). Intestinal permeability changes in acute gastroenteritis: Effects of clinical factors and nutritional management. J Pediatr Gastroenterol Nutr 8:466–473. 2498498 10.1097/00005176-198905000-00008

[32] Noone C, Menzies IS, Banatvala JE, Scopes JW (1986). Intestinal permeability and lactose hydrolysis in human rotaviral gastroenteritis assessed simultaneously by non-invasive differential sugar permeation. Eur J Clin Invest 16:217–225. 3089818 10.1111/j.1365-2362.1986.tb01332.x

